# Hemorrhagic Fever-Causing Arenaviruses: Lethal Pathogens and Potent Immune Suppressors

**DOI:** 10.3389/fimmu.2019.00372

**Published:** 2019-03-13

**Authors:** Morgan E. Brisse, Hinh Ly

**Affiliations:** ^1^Biochemistry, Molecular Biology, and Biophysics Graduate Program, University of Minnesota, St. Paul, MN, United States; ^2^Department of Veterinary and Biomedical Sciences, University of Minnesota, St. Paul, MN, United States

**Keywords:** arenaviruses, Lassa fever, host-virus interactions, innate and adaptive immunity, viral immunology, viral pathogenesis, host defense

## Abstract

Hemorrhagic fevers (HF) resulting from pathogenic arenaviral infections have traditionally been neglected as tropical diseases primarily affecting African and South American regions. There are currently no FDA-approved vaccines for arenaviruses, and treatments have been limited to supportive therapy and use of non-specific nucleoside analogs, such as Ribavirin. Outbreaks of arenaviral infections have been limited to certain geographic areas that are endemic but known cases of exportation of arenaviruses from endemic regions and socioeconomic challenges for local control of rodent reservoirs raise serious concerns about the potential for larger outbreaks in the future. This review synthesizes current knowledge about arenaviral evolution, ecology, transmission patterns, life cycle, modulation of host immunity, disease pathogenesis, as well as discusses recent development of preventative and therapeutic pursuits against this group of deadly viral pathogens.

## Introduction

Significant progress has been made in recent years to understand the role of modern means of travel in inadvertently exporting deadly arenaviruses from endemic regions, the basic biology of these viruses, their genomic evolution and modes of transmission and immune suppression, and the disease pathogenesis for which they are responsible. A detailed level of understanding of the viral life cycle, evolution, and interactions with the host's immune signaling pathways is necessary in order to design effective therapeutic and preventative measures against this group of deadly human pathogens.

## Arenaviral Classification, Evolution, and Ecology

**Figure 1:**
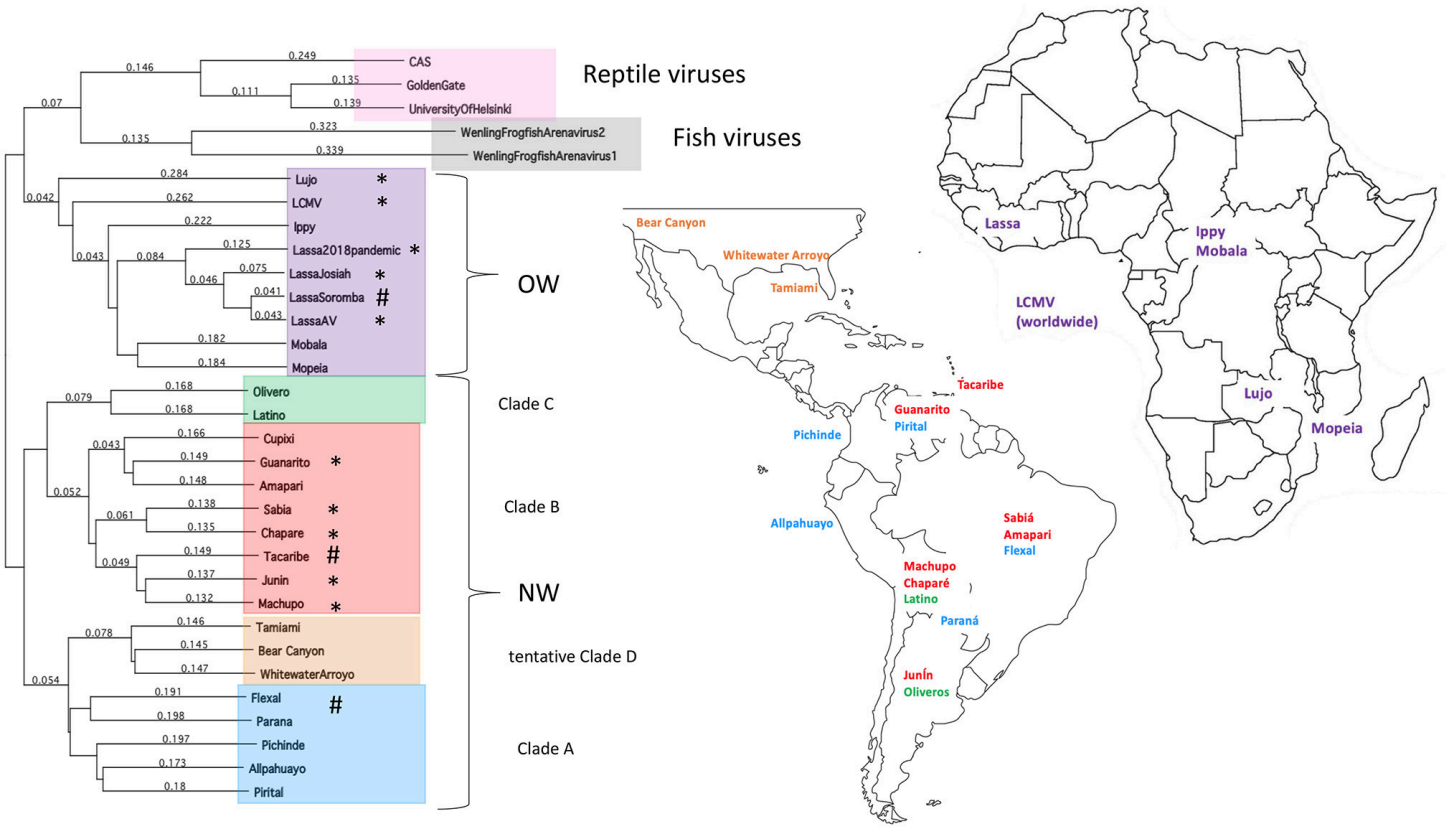
Taxonomy and location of arenaviruses. The phylogenetic tree for OW and NW arenaviral strains and their geographic locations. Tree was generated from full-length genomic sequences for the L polymerase protein aligned by Clustalw analysis. Asterisks designate strains that cause natural human diseases, whereas hashtags designate strains that can cause laboratory-acquired diseases in animals.

The family *Arenaviridae* is divided into 3 genera based on their natural hosts—*Mammarenaviridae, Reptarenaviridae*, and *Hartmaniviridae* that include viruses infecting mammals, reptiles, and fish, respectively (1, 2). *Mammarenaviridae* are further classified into the regions of their origins, such as the Old World (OW) viruses found in West Africa (3–9) and the New World (NW) viruses found in South America (5, 10–17), which are believed to have originated <23,000 and 41,000 years ago in those continents, respectively (18) (Figure 1). Additionally, several NW arenaviral strains have been discovered in the United States, which are suspected to potentially cause human disease (19, 20). The OW and NW subgroups are polyphyletic and contain both human-pathogenic and non-pathogenic viral strains, with 10 strains in total known to cause human diseases (21). Unlike other hemorrhagic fever viruses, such as the Ebola virus (EBOV), arenaviral transmissions to humans have been found primarily as a result of human interactions with the rodents as the natural reservoirs of these viruses, as has been directly observed as recently as the 2017–2018 Lassa virus outbreak in Nigeria (9). However, human to human transmission may play a larger role in certain viral outbreaks, such as a 2014 outbreak where strains across larger geographical areas were found to cluster closely together (22).

This transmission model presents an interesting challenge for determining the evolutionary history of arenaviruses. As with many zoonotic viruses that follow a co-speciation pattern to allow for infection of new hosts, arenaviruses have been previously thought to have originated in Asia along with the earliest rodents and later spread to Europe, Africa, and the Americas alongside the spread of the rodents (23, 24). However, arenaviral and rodent host phylogenetic trees almost never perfectly match (25) and some models result in rodent hosts that are randomly integrated into the arenaviral phylogeny (26). Additionally, only the Lymphocytic Choriomeningitis Virus (LCMV) has been found to circulate among European rodents (18, 27–30), but titers against LCMV have been found in human subjects worldwide (31, 32), suggesting that rodents and potentially other hosts for LCMV are more widespread than previously thought. Recent studies have revealed that LCMV can be isolated from ticks in the Ukraine (33) and in China (34) (though the low numbers of positively infected sample specimens and unknown capacity of LCMV to infect insect cells raise some doubts about ticks being a true reservoir rather than an intermittent viral carrier). Arenaviral evolution, therefore, may be more reflective of their adaptation to the available hosts based on geographic constraints (9, 18).

Local host adaption of arenaviruses is further evidenced by recent insights into diversity of arenaviruses in reptilian and marine hosts. The full genomic sequences have been determined for three reptarenaviruses isolated from boa constrictors and annulated tree boas: the CAS virus (35), Golden Gate virus (35) and University of Helsinki virus (36), necessitating the addition of the genus *Reptarenaviridae* into the *Arenaviridae* family in 2015 (1). These three reptarenaviruses have been found to be causative agents of Inclusion Body Disease (IBD), a fatal condition in snakes characterized by neurological abnormalities (37, 38), large eosinophilic aggregations inside infected cells (37–40) consisting of a 68 kDa protein (39) thought to be reptarenaviral nucleoproteins (NPs) (36, 41) and the primary source of lethality being secondary infections (38–40). Preliminary evidence has indicated that reptarenaviruses might have specifically adapted to boa constrictors, with viral replication being optimal at the reptilian 30°C and attenuated when grown at the mammalian 37°C condition (41), and infected boa constrictors having decreased rates of IBD and increased chances of becoming asymptomatic viral carriers than other snake species despite a high viral load (37, 38, 42). Like rodent reservoirs, reptarenaviruses have also been found to transmit vertically (43). Whereas, the genome of mammarenaviruses are bi-segmented with a small (S) segment and a large (L) segment, those of the reptarenaviruses are potentially more complicated, with some infected snakes carrying up to 4 different S segments and 11 different L segments per individual (44). Further adding to arenaviral genome diversity is the recent discovery of two new arenaviruses infecting ray-finned fish (2) with tri-segmented genomes, making them a potential intermediate between arenaviruses and their close “cousin” virus, the tri-segmented *Bunyaviridae* family found widely in arthropods (2, 45) that can also cause severe and lethal hemorrhagic infections in humans.

**Figure 2:**
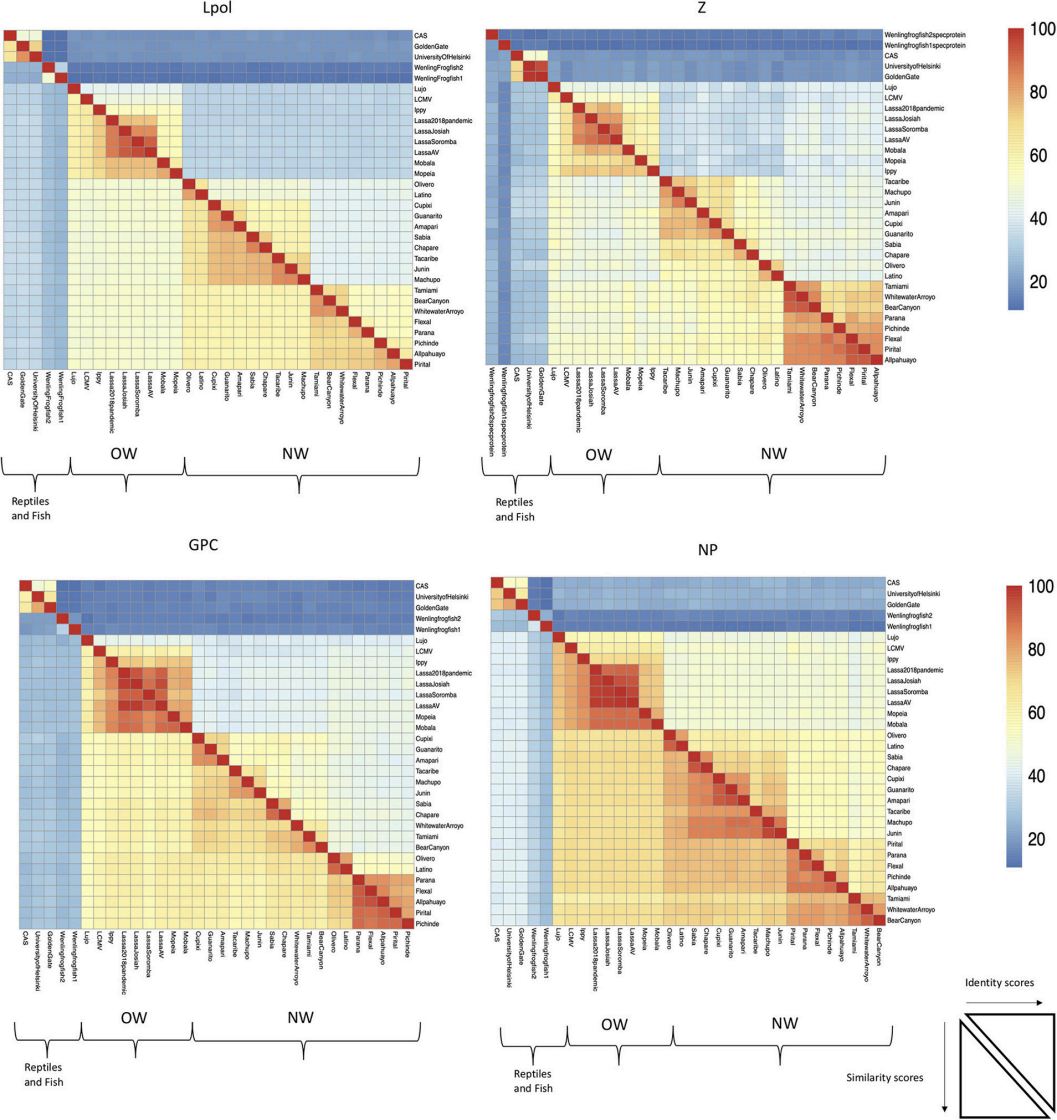
The L RdRp polymerase exhibits the least conservation among the arenaviral proteins. Full-length protein coding sequences from arenaviral strains were aligned by Clustlw analysis, and the matrix for pair-wise score similarity (# of shared amino acid residues/alignment length*100) was converted into a heatmap by the pheatmap module for R. The GenBank accession ID's used for alignments in Figures 1, 2 are as follows: NC_010249 (Allpahuayo L), NC_010253 (Allpahuayo S), NC_010251.1 (Amapari L), NC_010247 (Amapari S), NC_010255 (Bear Canyon L), NC_010256 (Bear Canyon S), JQ717261 (CAS L), JQ717262 (CAS S), NC_010563 (Chapare L), NC_010562 (Chapare S), NC_010252 (Cupixi L), NC_010254 (Cupixi S), NC_010759 (Flexal L), NC_010757 (Flexal S), JQ717263 (Golden Gate L), JQ717264 (Golden Gate S), NC_005082 (Guanarito L), NC_005077 (Guanarito S), NC_007906 (Ippy L), NC_007905 (Ippy S), NC_005080 (Junin L), NC_005081 (Junin S), HQ688674 (Lassa Josiah L), HQ688672 (Lassa Josiah S), MH888008 (Lassa 2018 pandemic L), MH887896, (Lassa 2018 pandemic S), FR832710 (Lassa AV L), FR832711 (Lassa AV S), KF478762 (Lassa Soromba L), KF478765 (Lassa Soromba S), NC_010760 (Latino L), NC_010758 (Latino S), AY847351 (LCMV L), AY847350 (LCMV S), NC_012777 (Lujo L), NC_012776 (Lujo S), NC_005079 (Machupo L), NC_005078 (Machupo S), NC_007904 (Mobala L), NC_007903 (Mobala S), NC_006574 (Mopeia L), NC_006575 (Mopeia S), NC_010250 (Olivero L), NC_010248 (Olivero S), NC_010761 (Parana L), NC_010756 (Parana S), NC_006439 (Pichinde L), NC_006447 (Pichinde S), NC_005897 (Pirital L), NC_005894 (Pirital S), NC_006313 (Sabia L), NC_006317 (Sabia S), NC_004292 (Tacaribe L), NC_004293 (Tacaribe S), NC_010702 (Tamiami L), NC_010701 (Tamiami S), KF297880 (University of Helsinki S), KF297881 (University of Helsinki L), MG599863 (Wenling frogfish 1 L), MG599864 (Wenling frogfish 1 S), MG59986 (Wenling frogfish 1 M), MG599866 (Wenling frogfish 2 L), MG599867 (Wenling frogfish 2 S), MG599868 (Wenling frogfish 2 M), NC_010703 (Whitewater Arroyo L), NC_010700 (Whitewater Arroyo S).

**Figure 3:**
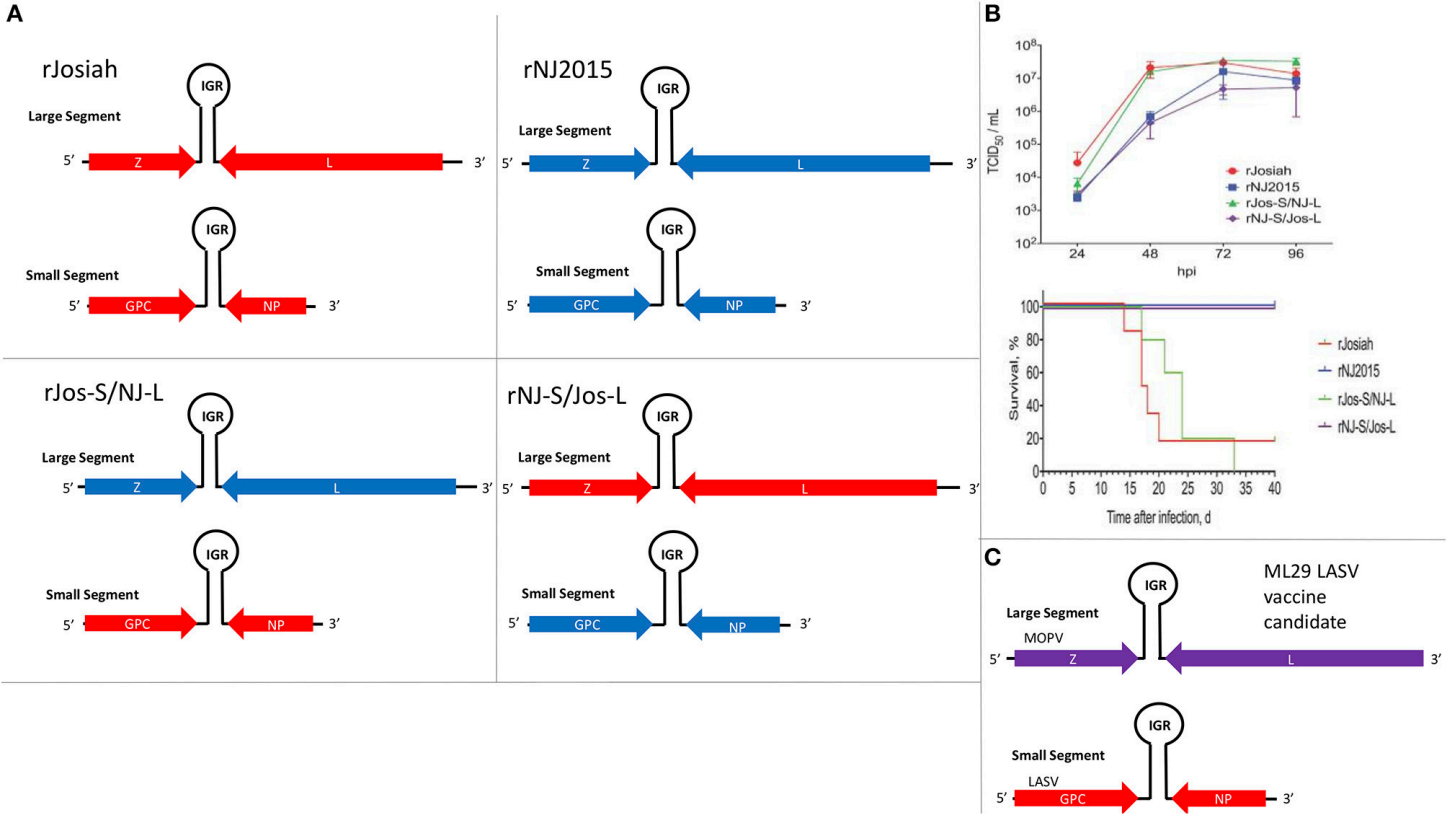
The S segment of the Lassa virus genome is sufficient to maintain pathogenicity. **(A)** Using reverse genetics strategy to produce recombinant wildtype (WT) and reassorted Lassa viruses carrying different large (L) and small (S) segments from different strains of the virus (Josiah vs. NJ2015). Viral genes: matrix Z, L RdRp, glycoprotein (GP), and nucleoprotein (NP); IGR, intergenic region. **(B)** Growth kinetics of viruses listed in 2A in A549 cells and the survival curve of strain 13/N guinea pigs subcutaneously infected with these viruses. Figure redrawn from Welch et al. (47). **(C)** Illustration of the genomic content of the ML29 LASV candidate vaccine carrying the L segment from the Mopeia virus (MOPV) and the S segment from LASV.

The focus of this article is on the OW and NW mammarenaviruses. Phylogenetically, changes in the viral polymerase of the mammarenaviruses may be more associated with older adaptive events dating to the speciation of NW arenaviruses (18), while more current adaptation, at least with the OW Lassa virus (LASV), appears to more prominently associate with the viral surface glycoproteins (46). This observation is recapitulated when comparing the genetic similarity of the four arenaviral proteins (Figure 2). The arenaviral polymerases have less homology between OW and NW viruses as well as among OW and NW strains compared to the other 3 arenaviral proteins, suggesting an earlier genetic divergence. A recent report further supports this observation by noting that a reassorted virus containing the S genomic segment that encodes the viral glycoprotein (GP) and nucleoprotein (NP) from the known virulent LASV Josiah and the L genomic segment that encodes the viral polymerase (L) and matrix protein (Z) from the known less virulent 2015/Liberia LASV strain retained the disease pathogenicity in guinea pigs (Figure 3). In contrast, another reassorted virus carrying the viral GP and NP from the 2015 Liberian strain was severely attenuated (47). Taken together, this suggests that earlier adaption to a geographically available host required fundamental changes to the internal viral proteins, such as the L polymerase, while more recent evolution is driven by optimizing viral entry efficiency and immune evasion mediated by viral GP and NP, respectively. The concept of viral proteins mediating host immune evasion will be discussed further below.

## Disease Transmission and Social Implications

Mammarenaviruses enter their hosts by inhalation of air-borne viral particles or by eating and/or drinking virus contaminated food or water, respectively. The social structures of arenavirus endemic regions present a particular challenge for viral containment with this transmission model. Homes are often open-air spaces and contain small spaces for rats to cohabitate with humans when built from locally available materials. As a result, rodent reservoirs move easily from home to home (48) and previous surveys have found significant numbers of homes containing rodent reservoirs and instances of contact with rodent or rodent waste products, particularly at night when the activity of rodents is at its highest level (49). Rice is often the stable crop in endemic areas (particularly in western Africa), which is typically grown in fields or low-lying swamps that encourage rodent habitation (50). The challenges presented by the educational and communication infrastructure in endemic regions also prevent optimal disease control, with a recent survey finding that 76% of residents in a Nigerian urban town had inadequate knowledge of Lassa fever and 51% had poor control practices (51). Practices such as hunting rodents for food and for use in sacrificial ceremonies have also been documented to increase risks for infection (50). Future measures of disease prevention will not only need to focus on enforcing individual habits of rodent control, but also on encouraging larger political and incentive policies conducive to foster sensible habits (52, 53) and infrastructural building, as diagnostic laboratory capable of carrying out viral genomic PCR to detect arenaviral infections is often not available in endemic regions (54–56). Computational modeling of regions at risk of arenaviral pandemics will also continue to be necessary as predictive/preventive measures (53, 57–60).

Several modeling studies have indicated that several African regions in close proximity to current endemic regions are at serious risk for arenavirus spread (53, 57, 58), and new reservoirs for human pathogenic arenaviruses are continuing to be found, adding to the potential for interregional spread (61). However, the biggest factor in restricting arenaviral pandemics appears to be a strong inter-species host transmission barrier (62). Current models suggest about 10% of rodents in endemic areas are seropositive for local arenaviruses, and that rodents clear the virus in a time period significantly shorter than their life span to produce antibodies (63, 64) with some preliminary evidence suggesting that anti-arenaviral antibodies may have a small correlation with decreased survival and increased rodent capture (65).

There is increasing concern about the potential for arenaviruses to spread across regions and initiate worldwide pandemics. LASV remains the only documented arenavirus to be imported by travelers who have visited endemic regions for a variety of reasons (Supplemental Table 1). While most documented cases occurred prior to 2000 (66), more recent cases of Lassa fever importation have been reported in the United States [Pennsylvania (67), Minnesota (68), and Georgia (69)], Ghana (70), Sweden (71), and Germany (72, 73). A common theme among these cases is that, while no secondary infections as of yet have been documented, an intermittent period of remission between initial treatment and reemergence of symptoms presents a potential risk for protracted disease and transmission (74). A salient fact that arenaviruses also have the potential to be used as biological weapons (75, 76) is of particular concern for endemic regions that are engaged in constant political and military conflicts (77). Human-to-human transmissions, although are rare occurrences, have been documented through the use of contaminated medical instruments in standard and specialized medical procedures, such as organ transplantations (78). As a whole, the threat of arenaviral spread and exportation from endemic regions should be of concern for public health considerations despite natural barriers that currently help curb major outbreaks.

## Arenaviral Life Cycle

### Entry Mechanisms by NW and OW Arenaviruses

**Figure 4:**
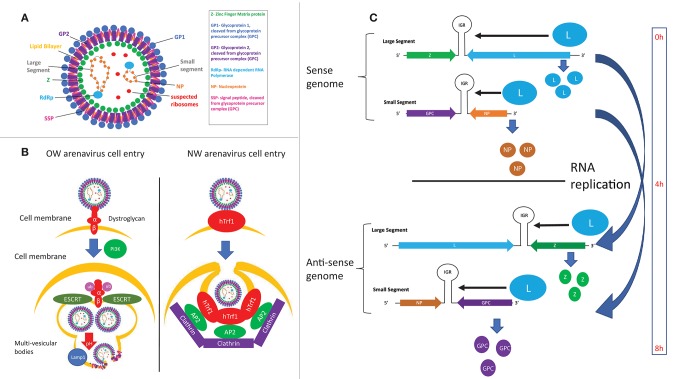
**(A)** Structure of arenaviral particles. **(B)** Comparison of the entry mechanism for OW and NW arenaviruses. **(C)** Timing of protein expression and RNA replication during the arenavirus lifecycle as determined by the genomic structure.

Arenaviruses are enveloped, ambisense single-stranded RNA viruses. Their structure consists of a membrane envelope containing the surface glycoprotein (GP) subunits surrounding a capsid, which consists of the Z matrix protein (Figure 4A). Inside the capsid are the L RNA-dependent RNA polymerase (L RdRp) and the viral bi-segmented genome encapsulated by the nucleoprotein (NP). Cell entry is mediated by GP, which is first expressed as the glycoprotein precursor complex (GPC). The host subtilase SK1-S1P proteolytically cleaves GPC into its three final subunits (79–81): GP1, GP2, and SSP (Stable Signal Peptide), which form heterotrimers on the cell membrane surface (82–84). GP1 forms spikes protruding from the viral envelope and is responsible for interacting with entry receptors, while GP2 is a class 1 fusion transmembrane protein by virtue of its 6 helix domain (85, 86). SSP (87) is an unusually small, hydrophobic and long-lived (88) signaling protein implicated in viral fusion (89, 90), transport of the GPC complex through the SK1-S1P containing Golgi (91) and as a GPC folding chaperone (92, 93). While recombinant arenaviruses expressing a SSP from a different strain are viable, recombinant viruses expressing a non-arenaviral signal peptide are not (94), indicating the specific adaptation of SSP for arenaviral proteins. This observation is strengthened by work from our laboratory, which shows that certain SSP residues attenuate viral growth independently of cell entry efficiency, illustrating the multiple roles of SSP in the viral life cycle that remain to be fully elucidated (95). GP1 and GP2 also contain N-glycosylation residues at multiple sites (96), while SSP is myristoylated (89). While the purpose of these post-translational modifications has yet to be described in detail, GP1/2 glycosylation has been linked to viral protein transport (96) and SSP myristoylation has been found to be vital for directing membrane anchor and viral fusion (89, 90).

LASV and LCMV were first discovered to utilize α-dystroglycan as their cellular entry receptor (97), which was later confirmed to be the case for all OW and clade C NW arenaviruses (98) (Figure 4B). Glycosylation of α-dystroglycan by the glycosyltransferase LARGE is necessary for recognition by arenaviruses (99–101), which mimics the interaction between α-dystroglycan and its natural ligand laminin (102). Regulation of α-dystroglycan appears to be a driving genetic force in arenaviral endemic regions, as wide-scale genomic studies have found evidence of positive selection of polymorphisms of LARGE and other α-dystroglycan regulatory proteins in Nigerian populations (103–105). The C-terminal GP1 subunit directly interacts with α-dystroglycan (97, 106–108), while the C-terminal GP2 subunit has been found to be critical for stabilizing the α-dystroglycan-GP complex (109, 110). Residue 260 on GP1 was the first residue found to be important for interaction with α-dystroglycan (98, 111–113). X-ray crystallography analysis later identified the loop 1 structure, and specifically residues 153, 155, and 190 of GP1, to be responsible for directly interacting with α-dystroglycan (107). Residues 260 and 136 were also found to be critical for cell entry, but these residues were both located up to 25 Angstroms away from loop 1. Given that full-length GP is required for cellular entry, it is theorized that these extra residues may facilitate cell entry by complex and unknown mechanisms involving the full length protein (107). Supporting this idea, residues at the trimeric interface of the GP were recently found to also be critical for cell entry (108), highlighting the importance of the full GP for efficient cell entry.

The endosomal cellular protein LAMP1 has been identified as a critical host factor for mediating a “pH-switch” mechanism of virus-cellular membranes fusion and endocytosis of the virion particle, which can partly explain previous observations that chicken cell lines are among the few not able to be infected by LASV as they lack LAMP1 expression (106, 114). Conserved histidine residues on the GP1 subunit among OW arenaviruses have been found to mediate recognition by LAMP1 (108), and structural analysis has found a shift in GP1 conformation following its interaction with LAMP1, possibly serving as an immune cloaking mechanism (115). Another interesting mechanism has also been proposed for LAMP1-mediated LASV endocytosis, where LAMP1 raises the pH threshold for acidic endocytosis, thereby increasing virus yield by preventing deactivation of the viruses by the acidic environment inside the endosomes (116). Additional host factors and possible alternative entry receptors have also been identified for entry of arenaviruses that might involve the sodium hydrogen exchangers (117) and the phosphatidylserine receptors Axl (118) and TIM-1 (119), which need further analyses (120). A recent study has also found that the cellular NRP2 factor and CD63 appear to serve as substitutes for α-dystroglycan and LAMP1 to mediate entry of the Lujo virus (LUJV) (121), implicating the conservation of similar mechanisms in other viruses as well as the importance of conserving these mechanisms during viral evolution.

NW arenaviruses use different cellular entry receptors depending on their clade. Clade B NW arenaviruses that are pathogenic in humans have been found to use human transferrin receptor 1 (hTrf1) (122, 123) while non-pathogenic clade B viruses use hTrf1 orthologs (124, 125). A number of other viral receptors and host factors have also been found to allow Junin virus (JUNV) entry, with hTrf1 being the major candidate cellular receptor (123), and DC-SIGN/L-SIGN (126), voltage-gated calcium channels (127), and proteins involved in the clathrin-mediated pathways such as the dyn2/Eps15 endocytic pathway (128) being possible co-factors. TRIM2 has recently been identified in a siRNA screen as an inhibitor of NW arenaviral cell entry by a yet undetermined mechanism (129). The receptors for the other clades of NW arenaviruses are still unknown, though it has been shown that the tentative clade D viruses do not use hTrf1 (130).

Viral particles enter cells by clathrin-mediated pathways for α-dystroglycan-independent NW arenaviruses (131), and a clathrin-independent mechanism that involves PI3K-mediated formation of multivesicular bodies during late endocytosis and the ESCRT sorting pathway for α-dystroglycan-dependent viruses (132) (Figure 4B). GP1 disassociates from the viral particles upon exposure to acidic pH, exposing GP2 residues prior to viral fusion (133, 134). Viral fusion then ensues, of which the hydrophobic regions in GP2 (109, 135) as well as the cytosolic tail (110) have been found to play a critical role.

### Viral Genome Replication, Gene Expression, and Assembly

Replication of the viral genome occurs in the cytosol following its release from the endosome. The viral NP and L proteins are the minimum known components required for viral genome replication and transcription (136–138), and the viral Z protein is an inhibitor of the viral polymerase's transcriptional initiation function (137–140).

The viral genomic RNA structural elements play important roles in the regulatory mechanisms of viral genome replication, transcription, and gene expression. The 5′ and 3′ untranslated regions (UTRs) of arenaviral genomes contain complementary sequences that are predicted to form panhandle structures and are required for effective viral RNA replication and transcription (141, 142). Specifically, 19 nucleotides located at the terminal 3′ UTR of the LCMV genome were found to serve as a minimally required promoter element and that the sequences located at the complementary 5′ UTR to form the panhandle structure appear to be required for efficient genome synthesis (142). Foscaldi and colleagues have reported that the 5′ UTR of the Tacaribe virus (TCRV) appears to contain translational stimulatory signal, whereas some sequences within the 3′ UTR can down-regulate viral gene expression (142, 143). The intergenic regions (IGRs) fold into hairpin secondary structures that are thought to help terminate transcription (12, 144, 145) and to protect the unique non-polyadenylated viral mRNA transcripts from degradation by the cellular exoribonucleases (145). These sequences appear to have a large tolerance for sequence variations that can be exploited for the development of attenuated vaccine candidates (21, 146, 147). It is noteworthy that during viral genome replication, genomic primers slip backwards from their initial binding site, resulting in a non-templated 5′ ppGpp residue (148) that is thought to act as a viral RNA decoy by competitively inhibiting the viral RNA sensing by the cellular innate-immune machinery (149). The molecular mechanisms of viral RNA sensing and innate immune evasion strategies by arenaviruses as well as on efforts to develop vaccines will be discussed in detail below.

The localization of arenaviral RNAs has also been found to be important for replication control, with both sense and anti-sense RNAs associating with cytosolic compartments containing viral NP and host factors involved in RNA metabolism (150). In this regard, recent advances in antibody-mediated dsRNA visualization (151) as well as specific probing for genomic and anti-genomic arenaviral RNA (152) show much promise for elucidating the dynamics of arenaviral RNA during the infectious life cycle. Preliminary evidence suggests that the timing of viral RNA replication is under regimented control mechanisms, as NP and L genes being transcribed and translated first and directly from the negative-sense viral genome, whereas GPC and Z are expressed several hours later after the viral genomic RNA segments encoding these genes have been replicated and then transcribed (152, 153) (Figure 4C). Additionally, virally infected cells appear to amplify viral genes in cyclical waves of expression and viral clearance, the molecular mechanism of which is unclear (152). Future studies in this area will include method development and optimization in order to image RNA species at lower copy numbers and to expand the RNA repertoire available for targeted imaging.

After the viral RNAs are replicated, transcribed, and translated, virion assembly ensues and is mediated by both viral and host cellular proteins. The C-terminal domains of the viral Z matrix protein is a central player for viral assembly, budding, and release from the infected cells. Z-L interactions (140, 154), Z-NP interactions (155), and Z-GP interactions (156, 157) have been shown to ensure co-localization of viral proteins for assembly. Z then interacts with the cellular Tsg-101 ESCRT pathway proteins to allow viral budding process to begin (158, 159), which is aided by a myristoylation residue at the N-terminus of Z to allow for interaction with the cellular membrane (160, 161). Z proteins has also been found to be capable of self-budding and the production of the viral like particles (VLPs) in the absence of the viral RNA or other proteins indicates that Z is both necessary and sufficient for budding (162–165). A recent report suggests that the late domains located in the C-terminal part of the Z protein may also be required for the release of the so-called defective interfering (DI) virion particles from the infected cells but not necessarily for the release of infectious virions, a process that may be regulated by phosphorylation of certain residues in the viral Z protein (163). DI particles are produced by many viruses, including arenaviruses, during infection and are similar to infectious virion particles in appearance and protein contents but they cannot produce productive infection (166) as their genomes contain large and deleterious deletions (167). Such deletions have been observed in the 3'UTR regions of the LCMV genome and potentially elsewhere in the viral genome, but their specific functions in inhibiting viral replication have yet to be studied in detail (168). Arenaviruses have been found to produce high levels of DI particles in cell culture (169) and in animal infection models (170), and DI particles have been theorized to contribute to viral persistence (166, 171, 172).

## Disease Pathogenesis and Host Immune Suppression

**Figure 5:**
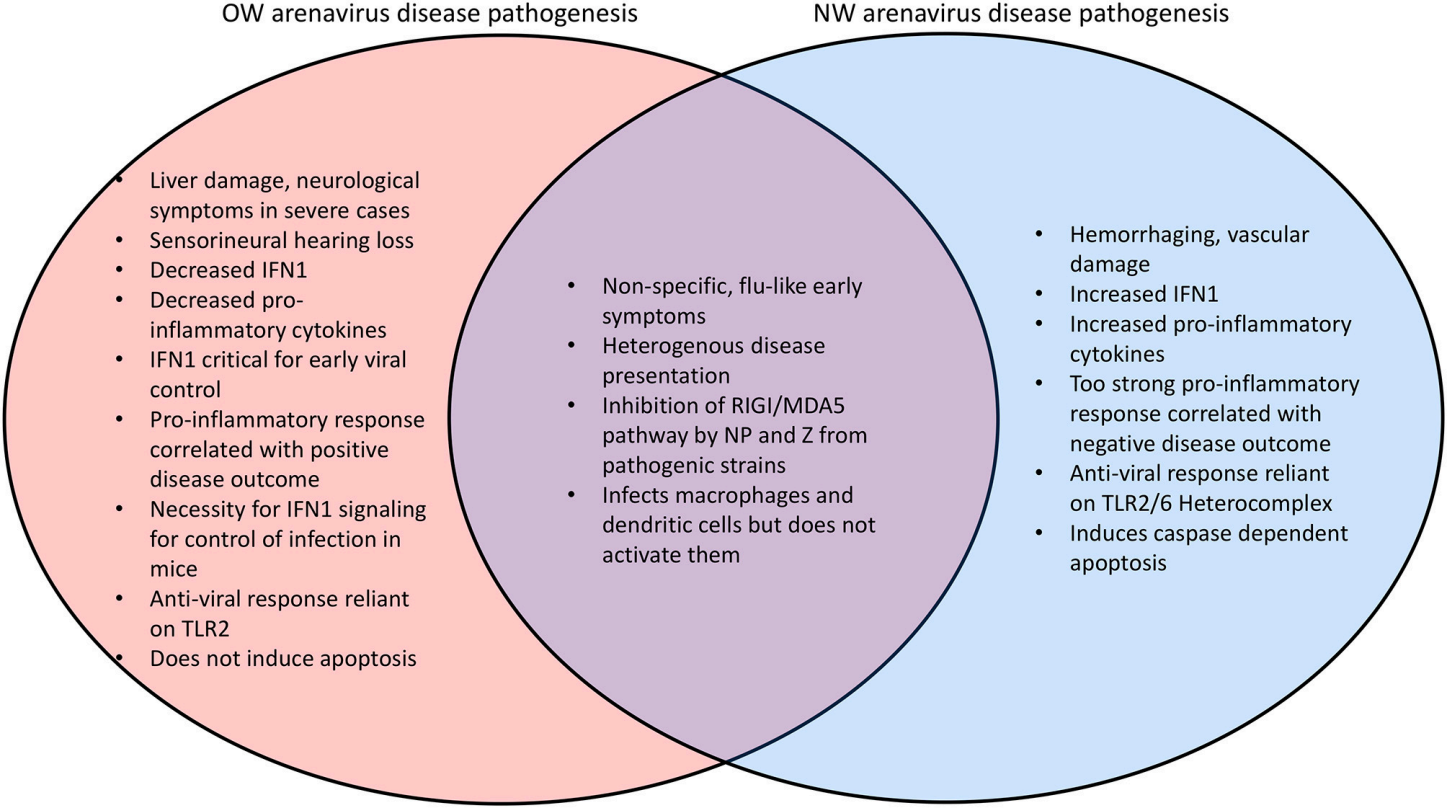
Comparison of the disease phenotypes between OW and NW arenaviruses.

Mammarenaviral disease profiles tend to be heterogenous (173–176). Lassa fever, the most severe arenaviral disease, is estimated to cause up to 300,000 cases and 5,000 deaths per year in endemic regions of West Africa (5, 173). A recent outbreak occurred in Nigeria has resulted in 431 laboratory confirmed cases that include 37 health care workers and with a 25% overall fatality rate (177–179). Previous outbreaks have recorded lower mortality rate in hospitalized patients (180). However, the fatality rate can be as high as 87% among infected women during the third trimester of pregnancy (181), and maternal-fetal transmission of arenaviruses has been demonstrated in rodent models (64) as well as in one recent reported case in humans (182). Many other complications arise from severe arenaviral infections including liver (183–186) and vascular (185, 187, 188) damage, both of which have been recapitulated in a guinea pig arenaviral animal model (189–191) as well as in immunocompromised mice (192, 193) and a novel hamster NW arenavirus model (194). While liver damage tends to be highly prominent in infections by OW arenaviruses with platelet factors and heme breakdown products as significant biomarkers of LASV disease, hemorrhaging, and vascular damage occur more frequently in NW arenavirus infections (195) (Figure 5). Sensorineural hearing loss (SNHL) also occurs in 29% of the survivors of Lassa fever (196–199) which may be attributed to a pathogenic inflammatory response in the auditory nervous system (200, 201). Other neurological complications have also been reported for arenaviral infection (202, 203).

Monocytic cells such as alveolar macrophages and dendritic cells are the earliest target for arenaviral cell entry. However, these cells are not activated upon viral entry, as evidenced by the lack of increase in the levels of activating markers, such as CD80, CD86, CD40, CD54, and HLAs as well as cytokines TNFα, IL1β, IL6, and IL12 (204, 205). For this reason, monocytic cells are thought to act as a viral “reservoir” early in infection, where the virus can easily enter, and virus later spreads when these cells enter draining lymph nodes (206, 207). The failure of these cells to become activated is consistent with the observation of the generalized immunodepression in severely and fatally infected individuals (208, 209). Virus replication has also been directly observed in a number of cell types, including respiratory epithelial cells following infection in human (210) and animal models (211).

**Figure 6:**
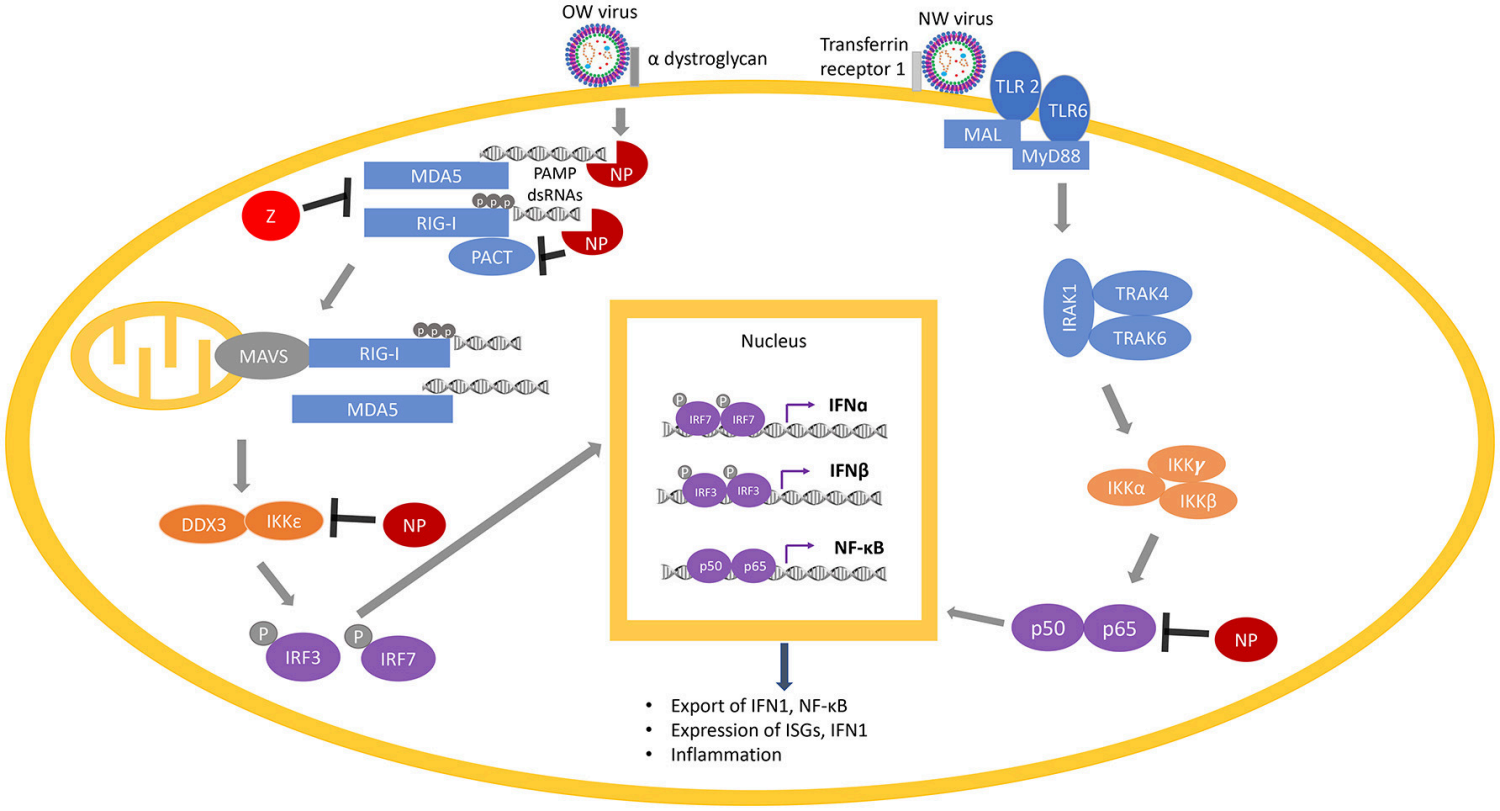
Arenaviral proteins (NP and Z) inhibit the RIG-I/MDA5 and NF-κB pathways. RIG-I and MDA5 are activated by PAMP dsRNAs during virus replication and are potentiated by PACT. Following the activation of MAVS (on the mitochondria) by RIG-I/MDA5, a molecular cascade involves the interaction of IKKε and DDX3, which is followed by phosphorylation of the transcription factors IRF3 and IRF7 to translocate them into the nucleus, where they dimerize and bind to transcription factor binding sites of the IFNα and IFNβ genes to activate their transcriptions. Expression and exportation of these gene products into the cellular milieu trigger the IFN1 signaling cascade in an autocrine or paracrine fashion to induce expression of hundreds of interferon-stimulated genes (ISGs) and inflammatory genes to confer virus resistance. The NF-κB pathway is activated by recognition of certain features of the virus particles by the membrane receptors TLR 2 or TLR 6. This initiates a molecular cascade resulting in the translocation of the two functional NF-κB units (p50 and p65) into the nucleus, resulting in more production of NF-κB. Arenaviral proteins (NP or Z shown in red) are known to inhibit different steps of the RIG-I/MDA5 and NF-κB pathways by either degrading the PAMP dsRNAs (through the NP RNase function) or directly inhibiting the normal function of different cellular proteins (RIG-I, MDA5, PACT, IKKε, or p50/p65).

Studies on human and rodent survivors of mammarenaviral infection have all indicated that proper functioning of the immune responses (innate and T-cell mediated immunity) are critical to minimizing viral growth rates, presentation of symptoms, and mortality rates (209, 212). The innate immune response is a compilation of non-specific defense mechanisms against foreign antigens that is critical for early detection and inhibition of pathogen growth before the adaptive immune response has time to produce proper cell-mediated immunity, such as the development of antibodies and cytotoxic T-lymphocyte responses (CTL) against the invading pathogen and/or the pathogen-infected cells (212). Innate immunity is enacted within a few hours of host recognition of a pathogen-associated molecular pattern (PAMP). The most common viral PAMPs recognized by immune cells are unique molecular features of the viral genome, such as 5′ triphosphorylated RNA and double stranded RNA (Figure 6). Pattern recognition receptor proteins (PRRs), such as the RIG-I-like receptors (RLRs) RIG-I and MDA5, are activated by PAMPs and result in cytokine expression and the activation and recruitment of innate immune cells such as macrophages, neutrophils, and dendritic cells (213, 214).

RIG-I and MDA5, upon binding to PAMP dsRNAs in the cytosol during the process of viral replication, change their conformations from a closed to an open and activated state (215). Activated RLRs (RIG-I and MDA5) initiate several complex molecular cascades, including activation of the mitochondrial anti-viral signaling protein (MAVS), which eventually results in the translocation of transcription factors such as interferon-regulatory factors (IRF) IRF3 and IRF7 as well as nuclear factor kappa B (NF-kB) into the nucleus of the cells to activate the expression of the type 1 interferons (IFN1) that include IFNα and IFNβ, and a wide variety of interferon stimulated genes (ISG) (216, 217). A recent study has suggested that IFNα may be primarily involved in inhibiting viral growth in early stages of viral infection, while IFNβ may control viral growth in the later stages of infection (218). IFN1 is then secreted and bound by their receptors (IFNAR) on the surface of either the same cell or the neighboring cells, which activates the IFN signaling cascade in a positive feedback loop to produce more of the IFN1 and the antiviral gene products in order to confer virus resistance (219, 220).

OW arenaviral infection (e.g., LASV) in patients with moderate to severe symptoms is associated with inhibition of the innate immune response with decreased levels of IFN1 and pro-inflammatory cytokines (184, 221, 222) (Figure 5). The importance for IFN1 signaling for control of arenaviruses appears to be conserved in mammals, as wildtype (WT) mice do not exhibit symptoms during arenaviral infection but IFN receptor (IFNAR) knockout (KO) mice have been found to succumb to disease (223, 224). Additionally, *in vivo* infection of non-human primates (NPHs) and guinea pigs with the LASV *Soromba* strain isolated from local rodents in Mali, Africa, shows a significant increase in cytokine signaling and decrease in mortality compared to other LASV strains (8). Mice infected with the OW LCMV, which is capable of maintaining persistent infection with low levels of IFN1 signaling (225, 226), display an IFN1 burst 6–48 h after infection and 2–4 days before a peak in viral titers, suggesting that IFN1 signaling is most important for inhibiting viral proliferation in the earliest stages of infection (209, 227). The difference between the immune responses in rodents and humans is one of the key aspects of active investigations. Rodent reservoirs generally do not experience severe symptoms from OW arenaviral infections (65, 228, 229), and mice must be immunodeficient to experience significant symptoms. LASV infected mice also experience an early upregulation of cell adhesion molecules in the peripheral blood mononuclear cells (PBMCs) consistent with immune cells being recruited to the site of infection (230). NW arenaviruses differ from OW strains in that they tend to produce increased levels of IFN1 and cytokines, and a pro-inflammatory response is favored for inhibiting viral proliferation (222, 231–234) (Figure 5). Virus-infected A549 cell cultures (235, 236) as well as fatally infected patients (237–239) have been shown to have consistently up-regulated levels of IFN1 and TNFα. However, macrophages infected *in vitro* with NW arenaviruses fail to show this upregulation (240).

The arenaviral NPs have been found to inhibit the RIG-I/MDA5 pathway. The crystal structure of the LASV NP and subsequent functional studies have revealed a 3′-5′ exoribonuclease domain located within its C-terminal domain (241–243) that preferentially degrades dsRNA (242, 244). The N-terminus of NP contains a unique domain that binds ssRNA (245) and has been proposed to bind to the m7GpppN mRNA cap structure (241). NP directly interacts with RIG-I and MDA5 (246), and recent work from our lab has demonstrated that NP prevents potentiation of RIG-I function by the cellular protein PACT and that the exoribonuclease domain of NP is required for this inhibition (247), the molecular mechanism of which will be discussed in detail in the next section. The exoribonuclease domain is also required for NP inhibition of the cellular protein kinase IKKε (248) and other cellular proteins in the IRF pathway (249, 250). Abrogation of the exoribonuclease domain has allowed for IFN1 expression in normally inactivated macrophages and dendritic cells (251, 252) and subsequent activation of natural killer (NK) cells resulting in antigen-presentation (253). The exception to NP-mediated IFN1 inhibition appears to be Tacaribe virus (TCRV) NP (254), which was initially thought to be due to variance in 4 exoribonuclease residues found in this viral protein (255). However, both structural and functional studies have later demonstrated that TCRV NP can effectively degrade dsRNA and inhibit the IFN1 pathway (244, 255). These studies collectively indicate the importance of PAMP dsRNA degradation by arenaviral NPs for inhibition of the IFN1 pathway.

One important consideration in determining the larger role of NP in arenaviral pathogenesis is the fact that IFN1 inhibition by NPs is also present in strains that are not known to be pathogenic to humans. For example, NP from the non-pathogenic Pichinde virus (PICV) has been found to inhibit the IFN1 pathway in human cells (254). Additionally, work from our laboratory has demonstrated that each of the 5 exoribonuclease (RNase) catalytic residues is necessary for IFN1 inhibition by Pichinde NP as well as for optimal PICV growth *in vitro* (256). RNase domain-mutant viruses have also been found to exhibit diminished viral load and pathogenesis *in vivo* in guinea pigs (which normally experience high lethality, develop human hemorrhagic fever-like symptoms and support a high viral load when infected by the P18 PICV strain), and even result in generation of WT revertant viruses (256), implicating the important role of NP RNase function for optimal viral replication. Altogether, the ability of NP to inhibit IFN1 expression by degrading PAMP dsRNA is likely conserved across known arenaviruses. As evidenced by the tight control of gene expression and viral RNA localization within cells, it is clear that regulation of viral RNA levels and localization is a critical mechanism of the virus life cycle to allow for maximum replication without being quenched early in the cycle by the innate immune response, which may explain the absolute conservation of IFN1 inhibition by arenaviral NP proteins.

Recent studies have also been focused on the role of the viral Z protein in inhibiting the IFN1 pathway. Originally, the Z proteins from 4 tested NW arenaviral strains [JUNV, Machupo virus (MACV), TCRV, and Sabia virus (SABV)] but not from the OW LASV or LCMV strains were found to directly interact with RIG-I and inhibit IFN1 expression (257). More recently, our laboratory has demonstrated that the Z proteins from both OW and NW arenaviral strains that are known to be pathogenic in humans can directly interact with RIG-I to inhibit IFN1 expression, but Z proteins from non-pathogenic arenaviral viruses cannot (258). These results have been recapitulated in primary human macrophages infected with WT and chimeric PICVs to show that the N-terminus of LCMV Z was sufficient to inhibit macrophage activation when expressed as a chimeric Z protein with the non-pathogenic PICV (259). As a whole, the Z protein is emerging as another key target in investigating the mechanism of innate immune suppression by arenaviruses, and an encouraging potential contributor to disease pathogenesis (i.e., a potential virulence factor) that will need further characterization (105, 260).

One area of study with increasing attention is the role of innate immune cells and cytokines in initiating the adaptive immune response against mammarenaviral infection. A key pattern that has emerged from preliminary studies is that an arenavirus's ability to suppress the innate immune response appears to be positively correlated with its ability to suppress any subsequent adaptive immune response. The correlation of an inhibited innate immune response with an attenuated and delayed T cell response in those succumbing to fatal arenaviral infection has been demonstrated in humans (210, 261, 262), non-human primates (221), guinea pigs (47, 190, 211, 263–265), immunodeficient mice (223, 224, 266) as well as in immunodeficient mice transplanted with cells from WT mouse bone-marrow (193). Accordingly, individuals that survive arenaviral infections manage to keep viral loads low, peaking 10–20 days after infection for LASV, while individuals that succumb have viral loads that continually increase exponentially (205, 221, 261, 262, 267–269). In WT mice, LCMV induces robust expression of key cytokines such as the type I and II IFNs as well as IL-18, the production of type II IFN being dependent on CD8 T cells (270). This not only illustrates the close inter-connectedness of the innate and adaptive immune responses, but also further illustrates how an active immune response is key to the natural resistance against arenaviral infection in mice. The importance of the innate-adaptive interface was also recapitulated in a recent co-infection model of LCMV and *E. Coli*, demonstrating that an LPS-induced inhibitory mechanism of NK cells significantly attenuated the LCMV specific CD8 T cell response and the vitality of the infected mice (271).

CD8 T cells have been found to be indispensable for clearing arenaviruses (221, 272–279), while CD4 T cell (277, 280) and antibody (184) responses have been found to be dispensable. In fact, a potential contributing factor toward arenaviral pathogenicity as well as the failure of most previous arenaviral vaccine candidates in protecting against pathogenic strains may be a skewed CD4+ T cell response. STAT1 KO mice were only able to survive LCMV infection when CD4+ T cells were depleted (266), suggesting that a lack of IFN signaling could somehow overwhelmingly favor lethal CD4+ T cell proliferation. The determination of the mechanism of CD4+ T cell lethality, whether it is simply due to less CD8 T cells being activated or through some novel mechanism of CD4 T cells, has yet to be determined.

Myeloid dendritic cells (mDCs) are vital constituents in the innate-adaptive immune response interface due in combination to their large population sizes (281), their robust activation of CD8 T-cells (282), and their production of IFN1 as a CD8 T-cell activation enhancer under certain conditions, such as during LCMV infection (283). Dendritic cells (DCs) are a key target of infection by LASV (204, 205), but like macrophages, they are not activated by the infection (204, 205). However, this feature isn't consistent among arenaviral strains, as demonstrated by the well-documented comparative model of mammarenaviral immunogenicity of LASV and the Mopeia virus (MOPV). Both are OW arenaviruses and share 75% amino acid identity and the *Mastomys natalensis* African rat as a viral reservoir (23). However, MOPV is non-pathogenic in humans and has been considered as a potential LASV vaccine candidate in non-human primates (284), and no human cases of MOPV infection have ever been recorded (285). Initial studies found that MOPV infection of monocyte-derived dendritic cells (MoDCs) induced moderate activation and significant CD8 T-cell proliferation in the MoDC/T-cell co-culture system (286, 287), whereas LASV infected MoDCs induced low activation and weak and delayed T-cell responses (204, 287).

The same group of researchers have more recently demonstrated that both LASV and MOPV could not replicate well in myeloid dendritic cells (mDCs) isolated directly from PBMCs, and as a result, induced very low levels of IFN1 production, and that LASV-infected cells could not activate T-cells in the mDC/T-cell co-culture system (288). However, MOPV was able to induce higher levels of IFN1 and secreted cytokines known for inducing T-cell activation than in LASV-infected cells in the co-culture system, though the involvement of monocytes and potentially other cell types present in the co-culture system as a result of impurity could not be completely ruled out. Regardless, the observation seems to corroborate earlier findings indicating that DCs are dispensable for T-cell activation during LCMV infection (289). Like LASV, LCMV is known to inhibit innate immune response as demonstrated in another study (290).

Using chimeric viruses carrying different genes from either LASV or MOPV on either the LASV reverse genetics (RG) or MOPV RG backbone, the same group of investigators have shown that LASV NP is the main determinant in suppressing IFN1 production by mDCs (288), which is consistent with the known ability of all known arenaviral NPs to degrade PAMP RNA via their encoded RNase domain and thereby suppressing IFN1 production (105, 241, 244, 247, 252–256, 291). Due to the fact that mDCs did not appear to support productive replication of LASV and MOPV, it was difficult to ascertain whether the IFN1 suppressive effect was due to the expression of viral proteins in the infected cells by the different chimeric viruses. Moreover, the data could not explain the differentiation of mDC phenotype in the mDC/T-cell co-culture system between LASV and MOPV chimeric virus infections. In an attempt to provide a potential explanation for this observation, the authors have posited that various efficiencies of the NP RNase domains of different arenaviruses to suppress IFN1 production may explain differences among arenaviruses to induce DC-mediated T-cell activation. However, this hypothesis needs to be formally tested.

A chimeric LASV with a substitution of its GP with that of MOPV has been found to significantly increase IFN1 production (288), which may reflect different mechanisms of viral detection based on the different innate immune receptors (106). As an example, the Toll-like Receptor TLR2/TLR6 heterocomplex has been found to be responsible for innate immune activation by recognizing the GP of the NW JUNV upon virus infection for robust induction of the innate (292) and adaptive (293) immune responses (Figure 6), while only TLR2 has been implicated in the anti-viral activity of OW arenaviruses (294, 295). JUNV and other NW arenaviruses have also been found to initiate caspase-dependent apoptosis (233, 296–298), while OW arenaviruses fail to induce apoptosis (204, 298) or induce significantly delayed apoptosis (299) (Figure 5). The relative contributions of pathways directly mediated by the specific cellular receptor and other viral signaling mechanisms needs to be further characterized in future studies.

Contrary to earlier findings (258, 259), the Z protein was not found in the LASV/MOPV chimeric virus model to exhibit a significant effect on immune suppression (288), which might be attributed to different infectious models being utilized. It is worth noting that the experiments with the chimeric viruses did not take into consideration the potential differential gene expression levels of the viral proteins in virus-infected cells under the context of the chimeric virus backbones. Additionally, as mDCs did not appear to support productive viral replication, it raised a question of whether any of the viral proteins were synthesized in the virus-infected mDC model (288). It is therefore difficult to definitively discern the roles of the individual viral protein in modulating antiviral immunity in this unique *in vitro* viral infection model (288).

## Modulation of Innate-Immune Protein Functions by Arenaviruses and Other Viruses

Another mammarenaviral-host interaction that has an impact on IFN1 production is that with the cellular protein PACT (Figure 6). PACT is a 313-aa protein that contains 3 conserved dsRNA binding motifs (dsRBMs) with dsRBM1 and 2 binding to dsRNA and dsRBM3 mediating activation of the dsRNA-dependent kinase PKR (300). PACT has been shown to activate RIG-I via interaction with the C-terminal repression domain of RIG-I and activates its ATPase function to potently enhance IFN1 production upon viral infection (301, 302). Even though PACT has initially been identified as a cellular binding partner and protein activator of PKR (303), activation of RIG-I by PACT does not require PKR (302). Likewise, while PACT has also been shown to interact with and stimulate the cellular Dicer protein (303, 304), Dicer does not appear to play an important role in PACT-mediated regulation of antiviral response (302).

While PACT and RIG-I pull down together in co-immunoprecipitation assays, it is less clear whether this interaction occurs strictly through protein-protein interactions or by both proteins associating with viral and/or cellular RNAs or some combination of both. The potential role of RNAs in activation of the cellular innate immune proteins (RIG-I and PACT) has been strongly implicated in recent findings that arenaviral NPs require an intact dsRNA binding domain as well as its 3′-5′ exoribonuclease activity to block PACT enhancement of RIG-I function (189–191) (Figure 6). A number of other viral nucleoproteins can block PACT enhancement of RIG-I function, which also requires an intact dsRNA binding domain (305–307), or in the case of herpes simplex virus 1 (HSV1), is independent of the RNA binding capacity of the nucleoprotein (308). Recently, our laboratory has shown that arenaviral NPs block RIG-I potentiation by PACT, and that the exoribonuclease domain is required (247). However, NP does not appear to negatively impact PACT/RIG-I direct interaction, suggesting that NP may inhibit PACT indirectly by degrading dsRNA that may be associated with the complex and is essential for its proper function. Taken together, it appears that most if not all known viral protein partners of PACT (as well as PACT itself and its cellular protein partner RIG-I) have RNA-binding properties, yet it still isn't entirely clear whether dsRNA binding is an absolute requirement to inhibit PACT-induced RIG-I activation. These studies illustrate the current lack of detail understanding of the roles of the different viral proteins, including arenaviral NPs, to inhibit PACT-mediated RIG-I activation, and the nature of the dsRNAs (viral, cellular, or both) that may intimately be involved in this process. Future studies into the exact role and molecular mechanism (s) of virus-host interactions via the PACT/RIG-I pathway can serve as an appealing pan-antiviral therapeutic target for development. Additionally, antivirals targeting PACT can influence PACT-PKR mediated NF-κB and p53 stress signaling (300, 309–316) to impact not only antiviral control but also other known cellular processes involved in cancer and other physiological and metabolic conditions.

## Development of Vaccines for and Antivirals Against Arenavirus Infections

Currently, the only clinically successful anti-arenaviral vaccine is the anti-JUNV Candid #1 strain, which is currently manufactured by the Argentinian government but is not being considered for large-scale use due to its limited target population (222) as only Argentina is endemic for JUNV infection. To generate Candid #1, a human viral isolate (317) was used to passage twice in guinea pigs followed by additional passages in suckling mice and cell cultures (318–323). A number of unique mutations in Candid #1 were originally thought to attenuate Candid #1 compared to its parental WT strain (323, 324), and one such mutation (F427I in the transmembrane domain of the GP2 subunit) was consistently found to be sufficient for attenuation (325, 326). While the mechanism for this attenuation has yet to be elucidated, it is thought that the mutation may affect viral fusion or maturation efficiency (325). The equivalent mutation (F438I) in Machupo virus (MACV) was also found to be attenuated in mice (327), suggesting that this attenuation mechanism may be highly conserved. Additionally, the presence of a mutation in the SSP subunit along with the F427I mutation in GP2 is thought to prevent the virus from reverting to its wild type sequence (326). It is tempting to assume that similar attenuation techniques can be applied toward the development of other arenaviral vaccines. However, they may be limited to the JUNV vaccine for several reasons. While the IFN1 pathway (particularly IFNβ) pathway and subsequent T-cell response have been found to be critical in controlling arenaviral infection in mice (193, 209, 223, 224, 231, 266, 270, 328), arenaviral-associated immunosuppression results in limited T-cell responses (184, 204, 221, 222, 279, 287). Furthermore, the JUNV GP has been found to contain fewer glycans than the GPs from other mammarenaviruses (320, 329), and glycan residues on the glycan-rich LASV GP has been shown to promote neutralization antibody evasion (330). This is strengthened by recent observations that anti-JUNV antibodies from infected patients can neutralize other JUNV strains but cannot offer neutralization against other NW arenaviruses (331).

Partly due to the aforementioned reasons, the development of effective vaccines against other pathogenic mammarenaviruses (besides JUNV) has proven to be much more challenging. An early attempt was to use gamma-irradiated LASV particles, which produced considerable humoral responses in NHPs but failed to protect against fatal LASV challenge (332). A similar theme has been noted across subsequent studies where adaptive immunity [and even cross-protective immunity (333)] has been induced in vaccinated subjects, but has failed to protect against lethal viral challenge. Novel reverse genetic strategies are being explored to overcome some of these obstacles, such as codon optimization to increase the expression of viral protein antigens (334), tri-segmented arenaviral vectors on LCMV (335–339) or PICV (340, 341) backbones to increase viral attenuation, and single-cycle arenaviral vectors (342, 343) to minimize immunosuppression through exponential virus replication.

Another method to produce candidate vaccine in development is based on a reassorted virus known as ML29, which contains the L genomic segment of MOPV and the S genomic segment of LASV (269, 291, 344–346, 418–420) (Figure 3C). It was determined that the LASV NP and GP antigen expressions from this reassorted virus were essential to provide strong protective immunity in rodents (e.g., guinea pigs) and NHPs (e.g., marmosets and Rhesus macaques) against lethal disease caused by LASV infection (344–347). DI particles produced by persistently infected cells with ML29 have also been found to interfere with the replication of LASV, MOPV and LCMV, and to induce strong cell-mediated immunity in STAT1 KO mice (348), implicating the potential for formulation of ML29 with its DIs toward the development of a pan-arenaviral vaccine. Other LASV candidate vaccines include those expressing LASV GP or NP antigens from the genome of different recombinant viruses, such as Vesicular Stomatitis Virus (VSV) (349, 350), vaccinia virus (351, 352), MOPV (291), Yellow Fever Virus 17D (353, 354), alphavirus replicons (355, 356), and inactivated rabies virus (357), all of which have been shown to exhibit various protective efficacies against LASV infection in different animal models. Other LASV vaccine development efforts, which are being supported by a non-profit international organization called Coalition for Epidemic Preparedness Innovations (CEPI), include those that are based on new vaccine platforms, such as a recombinant measles-virus-based vaccine (358) and DNA-based vaccine (359, 360). CEPI has also supported two other LASV vaccine candidates that are based on recombinant rVSV-based vectors. The first is a rVSV construct expressing the LASV GP antigen based on the same platform applied to express the EBOV GP as a vaccine antigen that was used in a ring vaccination trial in Guinea, Africa (361, 362). The second is another rVSV-LASV GP construct with a translocated VSV-N gene and a truncated VSV-G cytoplasmic tail designed for increased vaccine safety. Similar attenuated rVSV vectors expressing single or multiple EBOV GPs have been shown to be immunogenic in NHPs (363) and successfully protected NHPs against EBOV and Marburg virus challenge (364–366), but there are currently no published pre-clinical data to support the development of a Lassa vaccine based on this platform. CEPI has also recently awarded a contract for pre-clinical development of yet another LASV-GP construct on the backbone of a non-replicating simian adenovirus, but again no pre-clinical information is available about the suitability of this viral platform for Lassa vaccine development (367). It remains to be seen how well the CEPI-supported vaccines will perform in the coming years. A formidable challenge of Lassa fever vaccine development is the requirement for a development of predominantly T-cell-mediated mechanism of protection, as strong memory CD4+ T-cell responses to LASV NP and GP2 of different strains of LASV have been demonstrated in some survivors of LASV infection in Guinea (368, 369). This is consistent with observations that, while CD4+ T-cells can provide a partial protection of ML29-vaccinated animals against lethal LASV challenge, depletion of CD8+ T-cells completely abolishes protection (370). Additionally, NP-specific CD8+ T-cells play a major protective role in mice infected with LCMV (279).

In addition to vaccine development as a preventative measure, the discovery of anti-viral drugs is a burgeoning area of arenaviral research. Currently, the only anti-viral treatment clinically in use that is specific to mammarenaviruses is convalescent plasma therapy against JUNV (175). Standard anti-viral nucleoside analogs, such as Ribavirin and Favipiravir (69, 371–374) have seen moderate clinical success, but are only effective when given in the earliest stages of infection when symptoms are primarily non-specific (173). Peptide-conjugated morpholino oligomers have also been tested as alternative nucleoside analogs, reducing the titers of several arenaviruses in cell culture and LCMV-infected mice (375). Nevertheless, recovery has been documented with symptom management in place of nucleoside analogs due to a delay in diagnosis being reached after the resolution of symptoms (376), limiting the desire for using nucleoside analogs as anti-viral compounds.

Recent advances in anti-arenaviral drug discovery have focused on identifying compounds that specifically target viral proteins or that modulate the activity of host proteins (377). The bulk of arenaviral drug discovery has focused on small molecule inhibitors of the mammarenaviral glycoprotein and cell entry, which has seen some successes with several different approaches. Amphipathic DNA polymers have been found to block LCMV GP-α-dystroglycan interaction by the virtue of its hydrophobicity rather than nucleotide sequence (378). Clotrimazole derivatives, which traditionally target the calcium-activated potassium channel KCa3.1, have also recently been found to inhibit arenaviral membrane fusion (379). However, its mechanism of action was found to be independent of KCa3.1, and thus currently unclear.

Conversely, a number of other compounds have been found to stabilize the GP-α-dystroglycan prefusion complex formation, thereby blocking pH-mediated endocytosis (380–384). One of these compounds (ST-193) has been found to reduce LASV titers in a guinea pig infection model (385). Another small molecule (LHF-535) has recently been found to inhibit a wide variety of arenaviruses with the exception of strains that contained a V434I mutation, which corresponds to the mutation responsible for attenuation in Candid #1, suggesting that it could also affect the prefusion stability complex (386). Finally, it has also been shown that a small molecule inhibitor of the cellular site 1 protease (PF-429292) can block GP processing (387, 388) as well as can serve as a general antiviral by inhibiting lipid and cholesterol synthesis needed for virus replication (389–392).

Some pharmaceutical compounds have also been found to disrupt the normal functions of mammarenaviral proteins. Given that myristoylation is necessary for Z-mediated arenavirus budding (160, 161), compounds that inhibit myristoylation enzymes (161, 393) have been found to change the cellular localization of Z and therefore inhibit virion budding from the infected cells. Aromatic compounds targeting the zinc-finger motifs of Z have also been found to inhibit arenaviral proliferation (394, 395). A variety of mechanistic effects have been characterized for one of these compounds. For example, the NSC20625 compound has been found to induce Z to unfold and accumulate in oligomeric structures (394) and thereby blocking the interaction of Z with the host PML protein and allowing nuclear bodies to form (396). The arenaviral L polymerase and NP have arguably experienced the least progress as drug targets. For the former case, one appealing potential pharmaceutical target is the cap-snatching mechanism of arenaviral polymerases. A similar drug has already been demonstrated to reduce titers of the West Nile virus (397). The specificity of viral cap snatching mechanisms (398) and the suspicion that arenaviral cap snatching mechanisms may be conserved across species (399, 400) make cap snatching a promising therapeutic target for anti-viral drug development (401). Some metal chelators have indeed been found to potently inhibit the LCMV L endonuclease domain and its subsequent cap snatching activity, demonstrating the potential for future antivirals targeting this virus feature (402). Lethal viral mutagenesis has also been identified as a possible mechanism for viral control (403, 404). While few studies have found drugs that target arenaviral NPs, one recent paper has identified a compound from a pyridine library that can reduce LCMV titers post-cell entry and is thought to affect NP and Z by unknown mechanisms (405).

Drugs that target host proteins are also being explored as potential pharmaceutical therapeutics. The tyrosine kinase inhibitor genistein is currently one of the most supported compounds, as ATF-2 phosphorylation has been found to be critical for viability of the NW PICV, which is susceptible to genistein *in vitro* (406, 407) and *in vivo* (408). Larger scale drug screens seem to implicate cellular kinase inhibition as a promising anti-viral target in general, with compounds affecting arenaviruses at multiple stages of the virus life cycle (409–411). Inhibitors of nucleoside production have also been tested in a variety of *in vitro* contexts (412–414) and may have additive effects when used in conjunction with nucleoside analogs (412). While these compounds didn't appear to negatively affect the vitality of cell cultures, they should be cautiously explored due to possible adverse effects on some rapidly growing cells *in vivo*. Finally, it may be possible to target immune signaling proteins as anti-viral control, with one anti-TLR inhibitor showing limiting LCMV-initiated cytokine responses in cells and viral replication in mice (294). For all potential anti-viral drugs, severe side effects (e.g., in neurological and embryonic development) due to known cytotoxicity of these compounds can be a great impediment to the widespread application of these compounds in treating arenavirus infections. Future anti-arenaviral drug and vaccine developments are still urgently needed to combat these deadly human pathogens.

## Summary and Future Directions

It is important to reiterate the important roles of the IFN1 pathway and T-cell-mediated immunity in controlling mammarenavirus infections (209). These pathways are also known to be critical for both the innate and the adaptive immune responses to other RNA virus infection models (415, 416) as well as during vaccination (417), further illustrating the importance for future studies of each of these mechanisms separately and in concert. In addition, continuation of ecological efforts to characterize the phylogeny and spread of arenaviruses (2, 9, 18, 21, 46), socioeconomic efforts to increase the public's awareness and capacity to contain disease (49–53), and vaccine and therapeutic developments are needed in order to optimize preventative and control measures for mammarenaviruses.

## Author Contributions

MB and HL contributed to the literature review and writing of the manuscript. MB prepared all Figures and Table.

### Conflict of Interest Statement

The authors declare that the research was conducted in the absence of any commercial or financial relationships that could be construed as a potential conflict of interest.

## Supplementary Material

Supplemental Table 1 Summary of documented cases of international importation of Lassa fever.
